# Transcriptional Profiling of Ligand Expression in Cell Specific Populations of the Adult Mouse Forebrain That Regulates Neurogenesis

**DOI:** 10.3389/fnins.2018.00220

**Published:** 2018-04-20

**Authors:** Kasum Azim, Rainer Akkermann, Martina Cantone, Julio Vera, Janusz J. Jadasz, Patrick Küry

**Affiliations:** ^1^Department of Neurology, Neuroregeneration, Medical Faculty, Heinrich-Heine-University, Düsseldorf, Germany; ^2^Laboratory of Systems Tumor Immunology, Department of Dermatology, Friedrich-Alexander-Universität Erlangen-Nürnberg, Erlangen, Germany

**Keywords:** neural stem cells, subventricular zone, neurogenesis, gliogenesis, growth/trophic factors, ligands, morphogens

## Abstract

In the adult central nervous system (CNS), the subventricular zone (SVZ) of the forebrain is the largest and most active source of neural stem cells (NSCs) that generates mainly neurons and few glial cells lifelong. A large body of evidence has shed light on the distinct families of signaling ligands (i.e., morphogens, growth factors, secreted molecules that alter signaling pathways) in regulating NSC biology. However, most of the research has focused on the mRNA expression of individual or few signaling ligands and their pathway components in specific cell types of the CNS in the context of neurogenesis. A single unifying study that underlines the expression of such molecules comprehensively in different cell types in spatial contexts has not yet been reported. By using whole genome transcriptome datasets of individual purified cell specific populations of the adult CNS, the SVZ niche, NSCs, glial cells, choroid plexus, and performing a bioinformatic meta-analysis of signaling ligands, their expression in the forebrain was uncovered. Therein, we report that a large plethora of ligands are abundantly expressed in the SVZ niche, largely from the vasculature than from other sources that may regulate neurogenesis. Intriguingly, this sort of analysis revealed a number of ligands with unknown functions in neurogenesis contexts that warrants further investigations. This study therefore serves as a framework for investigators in the field for understanding the expression patterns of signaling ligands and pathways regulating neurogenesis.

## Introduction

In the adult brain, the subventricular zone (SVZ) of the lateral ventricles is one of the main reservoirs of adult neural stem cells (NSCs) that generate new neurons and glial cells throughout life. SVZ-NSCs are present in quiescent phenotypes (qNSCs) and upon activation form activated NSCs (aNSCs) that give rise to transiently amplifying progenitors (TAPs) that will generate neurons via a highly migratory neuroblast stage. Newly generated neuroblasts migrate away from the SVZ along the rostral migratory stream and differentiate into interneurons mainly, and also a few glutamatergic neurons (Doetsch et al., 1999; Quiñones-Hinojosa et al., 2006; Brill et al., 2009). Depending on the transcriptional and signaling cues, SVZ-NSCs will additionally generate oligodendrocytes and astrocytes that invade the surrounding parenchyma (Menn et al., 2006; Benner et al., 2013), suggesting that there may be specialized subtypes of lineage restricted NSCs.

The SVZ is located around a spatially complex ventricular system and consists of a heterogeneous cell population, as a number of studies have identified that specific neuronal and glial subtypes are generated from discrete domains of the SVZ throughout life (reviewed in Fiorelli et al., 2015; Azim et al., 2016). This implies that regionally segregated NSCs are primed in a time-controlled manner for the generation of glial and neuronal subtypes, proposing that transcriptional mechanisms are additionally modulated by external stimuli that guide NSC fate. Such add-on regulators include microRNAs, exosomes containing proteins or RNAs, extracellular matrix constituents, additional and soluble secreted factors reviewed in Wakabayashi et al. (2014), Batiz et al. (2015), and Faissner and Reinhard (2015), the latter being one of the most studied in the field.

Identifying the source and expression patterns of signaling ligands with their role in NSC biology represents an important step in developing innovative strategies for manipulating germinal regions in health and disease. A number of earlier studies have described the expression patterns of signaling ligands by classical *in situ* experiments. For example in studies of young adult rodents, bone morphogenetic proteins (BMP4/7) were detected in glial-like cells in the vicinity of the SVZ (Peretto et al., 2004). Another classical ligand, epidermal growth factor (EGF) is expressed and secreted at somewhat distant sources from the SVZ, i.e., the striatum (Lazar and Blum, 1992). Other key ligands such as FGF2 have been shown to be expressed in the SVZ (Frinchi et al., 2008; Azim et al., 2012), whereas Shh is uniquely transported to ventral regions of the SVZ by axons projected from the ventral forebrain (Ihrie et al., 2011). The vasculature has been considered as a trophic source for maintaining or expanding NSC phenotypes (Thored et al., 2007; Tavazoie et al., 2008; Ottone et al., 2014; Crouch et al., 2015), but the expression levels of vascular-derived ligands in respect to other cell types are not fully understood. Moreover, additional secreted ligands are dispersed by the cerebral spinal fluid (CSF) (Thouvenot et al., 2006; Marques et al., 2011) and its flow is a major determinant of neurogenesis/gliogenesis (Silva-Vargas et al., 2016). Although these studies have given vital insights into some ligand expression, a thorough and systematic investigation of ligands across a variety of cell types has not yet been reported.

The increasing availability of whole genome transcriptome (WGT) datasets consisting of purified cell types of the young adult forebrain and of cells that constitute the SVZ niche, facilitates meta-analyses for the description of large scale expression patterns of signaling ligands involved in neurogenic processes. To this end, we present data analyses performed by focusing exclusively on ligands across multiple datasets that include adjacent glial cells, choroid plexus, NSCs and cells that comprise the SVZ niche (i.e., vasculature cells, ependymal cells, TAPs and neuroblasts). A number of region specific hallmarks in ligand expression were found and its transcriptome landscape could be mapped with enriched ligands presented for further future analysis. Most notably, a novel finding was that endothelia expressed a large number of transcripts, including key ligands the expression of which was apparently more abundant than previously thought. Intriguingly, data generated revealed a number of ligands that have not yet been studied in the context of adult neurogenesis/gliogenesis and warrants further studies. Altogether, these findings will aid investigators in the field by providing expression levels of key genes and families across a variety of cell types and may promote future functional insights.

## Methods

### Datasets incorporated into the study

Mouse Affymetrix datasets that have been reposited in GEO Pubmed (GSE numbers), representing NSCs of different subtypes (GSE54653) [postnatal day (P) ~70] (Codega et al., 2014); neural precursors (NPs)\TAPs, neuroblasts and ependymal cells (GSE18765) (P60) (Beckervordersandforth et al., 2010); oligodendroglia at different stages of development (GSE9566) (P16) (Cahoy et al., 2008); endothelial pericytes from the cerebral cortex (GSE47067; GSE48209) (~P70) (Nolan et al., 2013; Coppiello et al., 2015); (GSE29284) (Olson and Soriano, 2011); choroid plexus (GSE82308) (P60) (Silva-Vargas et al., 2016); astrocytes (GSE35338) (P30-35) (Zamanian et al., 2012); microglia (GSE58483) (~P60) (Israelsson et al., 2014) have been used. Of note, datasets of subtypes of neurons from different mouse forebrain regions that project axons and innervate the SVZ, have been generated using different platforms and were therefore excluded in this analysis in order to minimize any false positives. Datasets were processed for Robust Multi-array Average (RMA) normalization on CARMA software suite (https://carmaweb.genome.tugraz.at/carma/) comprehensive R- and bioconductor-based web service for microarray data analysis (Rainer et al., 2006), and additionally via R/Bioconductor using standard procedures. The Benjamini and Hochberg false discovery rate (FDR) was used to calculate *p*-values. The final gene lists contain all genes with FDR of 5% and >1.8 fold changes. Genefilter, heatmap.2 method implemented in the gplots, and pcamethods package was used for generating gene lists, and principle components analysis (PCA) using the statistics software R (R Development Core Team, 2008). The Venn diagram was made using http://www.interactivenn.net. Previous gene lists representing essential signaling ligands were used for further filtering parameters (Azim et al., 2017). All imported datasets were quality checked on a PCA plot for control- and housekeeping genes in discriminating overlapping properties and discarding datasets that were distant in the plot. The function of individual genes was classified using the resource http://www.genecards.org.

An analysis was performed for over-enrichment within these categories: (1) autocrine/paracrine signaling of NSCs (qNSCs; aNSCs); (2) adjacent glial ligand expression [microglia, astrocytes, mature oligodendrocytes (mOLs) and oligodendrocyte progenitor cells (OPCs)]; (3) secretion from the choroid plexus and (4) secretion from the niche, i.e., pericytes, endothelia, ependymal cells, neuroblasts and TAPs. Between these four groups, an enrichment analysis was performed. For example, for comparing expression profiles enriched in autocrine/paracrine NSC signaling, these datasets were compared with the remaining three categories. Similarly, this was then also done for enrichment for the remaining three groups. In this analysis, the four resulting gene lists were processed for further analysis and heatmaps were assembled applying default criteria. The cells that form the niche, i.e., endothelia, pericytes, neuroblasts and TAPs were grouped together as reported by other studies (Kokovay et al., 2010; Ottone et al., 2014; Crouch et al., 2015; Fiorelli et al., 2015). Ligand mRNA from endothelia and pericytes overlapped considerably, and the same was evident for neuroblasts and TAPs that otherwise expressed fewer ligand transcripts.

### Secretome pathways

The four different categories of ligands were further studied for functional analysis enrichment using the freely available Reactome (defined here as secretome) database using the latest version of the mouse-annotated database (Pathan et al., 2015). Using default settings, data was exported for each group and sorted by their *p*-values. Pathways with overlapping sets of genes were processed by taking the most significant in the list. In general, the minimal numbers of genes studied per pathway was “2,” except for those of the NSC category which a few obtained were only “1” and are stated with double asterisks in graphs. The top 10 are presented in order (clock-wise) of their significance and pathway terms shortened to fit in pie charts constructed using the genes associated with pathway terms in the data. Any obtained pathways with *p*-values (Hypergeometric test) >0.01 were discarded. For evaluating the pathways more enriched according to the genes populating the four groups we used ClueGO, a plugin of the freely available software Cytoscape (http://www.cytoscape.org). Each group of genes was represented as a separate cluster, and we performed an analysis by setting the same set of parameters and ran a pathway enrichment analysis of all genes' groups together. For graphical representation purposes, differences among the enrichment analysis of the single cluster were selected.

## Results

### A road map of signaling ligand expression in the mouse forebrain

**Figure 1:**
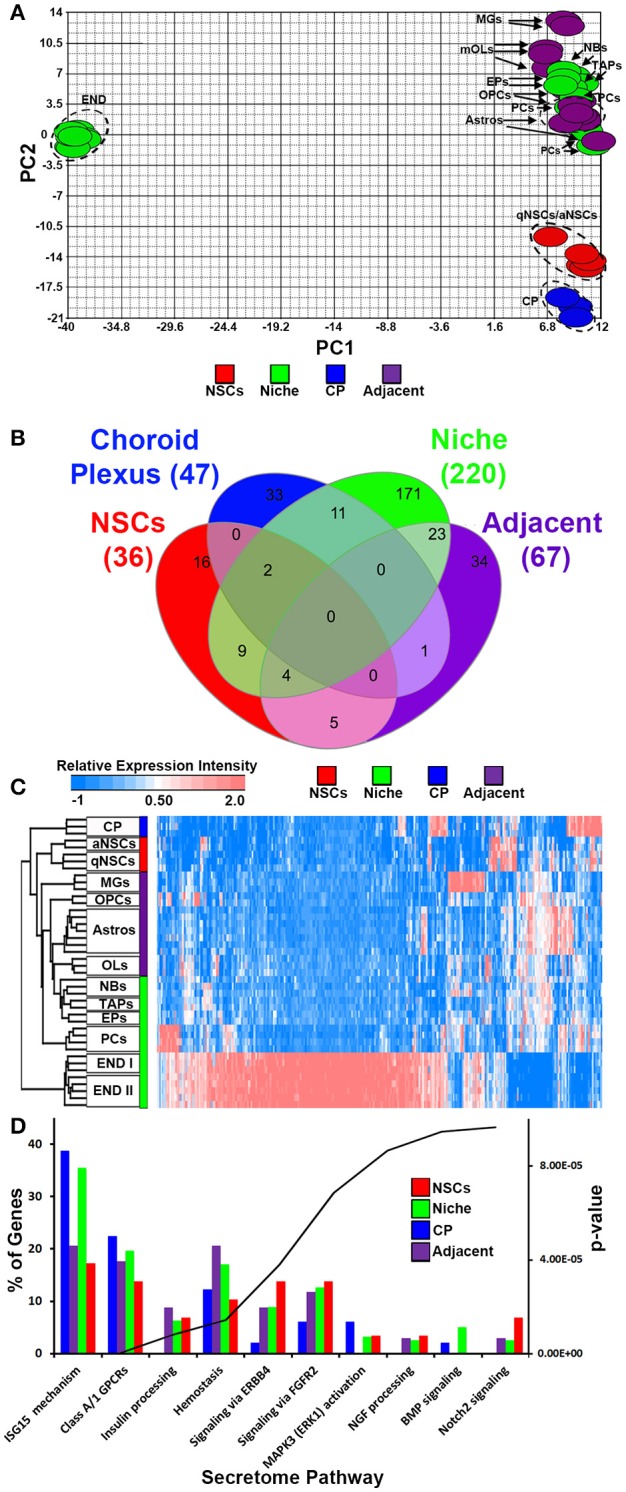
Ligand mRNA heterogeneity in purified cell populations of the adult mouse forebrain. **(A)** PCA ordering of ligands exclusively and significantly (*p* < 5% FDR) enriched throughout the different cell populations. Colors show the groupings of different cell populations. **(B)** Venn diagram of the 4 different groups displaying overlaps and specificity of significantly enriched genes (1.8 fold change; *p* < 5% FDR). **(C)** HC heatmap of all significantly enriched ligands in the analysis. **(D)** The ligand genes of the 4 different groups were classified using Reactome Pathway Analysis in ClueGO. The top 10 pathways (out of 17 obtained) were plotted and ranked according to their *p*-values. Data are shown as a percentage of ligands per pathway term. Red, green, blue and purple colors used are for groups containing NSCs, niche, choroid plexus and adjacent glial cells respectively. CP, choroid plexus; aNSCs, activated neural stem cells; qNSCs, quiescent neural stem cells; MGs, microglia; OPCs, oligodendrocytes precursor cells; Astros, astrocytes; OLs, oligodendrocytes (mature); NBs, neuroblasts; TAPs, transiently amplifying progenitors; EPs, ependymal; PCs, pericytes; END I, Endothelia 1; END II, Endothelia 2.

We incorporated and cross normalized a number of whole genome transcriptome datasets that have been generated from earlier studies using the same Affymetrix platform and at comparable ages. Using these datasets we performed an analysis to examine transcripts that are significantly altered across these groups taking into account recent gene lists of exclusively secreted signaling ligands, inhibitory signaling molecules, morphogens and trophic factors (Azim et al., 2017), resulting in 310 probes in total that were ≤ 5% FDR. Four groups for comparison were generated consisting of (1) qNSCs and aNSCs, termed as “NSCs,” (2) microglia, astrocytes and oligodendroglia termed as “adjacent,” (3) choroid plexus (CP), (4) endothelia, pericytes, TAPs, neuroblasts and ependymal cells, termed as “niche” (Mirzadeh et al., 2008). In cases where transcripts were represented by more than one probe, only the probe with the highest FDR value was analyzed further. Firstly, PCA was done against significant probes (Figure 1A), which designated all glial cells, pericytes, TAPs and neuroblasts exhibiting similar hallmarks, whereas qNSCs and aNSCs as well as the CP were unrelated. Surprisingly, endothelial cells showed dramatically distinct and distant covariance in their expression signatures. The four groups were compared with each other and plotted as Venn diagram (Figure 1B) revealing various degrees of overlaps. Intriguingly, ~60% of all ligand transcripts were derived from the niche, followed by adjacent (18%), CP (~12.5%), and NSCs (~9.5%), revealing that the niche itself is likely the most important signaling determinant in regulating neurogenesis/gliogenesis. All transcripts significantly different in this analysis were plotted on a HC heatmap which illustrates that the largest and overwhelming source of ligands are endothelial cells in the forebrain (Figure 1C). A gene ontology “secretome” (Reactome pathway) analysis was performed subsequently on these four groupings (niche, adjacent, CP, and NSCs) to examine gross differences amongst the genes related as % of the total genes expressed by different regions. In this analysis, potential sharing in regulated pathways amongst the four groupings were analyzed. Indeed, many of the ligands overlapped with ligands that target class A/1 (G protein coupled receptors) GPCRs, hemostasis (i.e., vascular-like and anti-inflammatory) and ligands that target Erbb4 receptors being common to all with the exception of the CP. But then some ligand expression patterns were rather unique. For example, ligands associated with ISG15 mechanisms (antiviral and immune-like pathways) were enriched in both the niche and CP (Figure 1D). Others, such as ligands targeting BMP and Notch2 signaling were upregulated in the niche and NSCs, respectively (Figure 1D). Altogether this implies that certain ligands detected in the four different groups exhibit a degree of redundancy whereas some ligand signatures show an extent of exclusiveness to a particular region or cell type, i.e., NSCs vs. the niche.

### Signaling ligand enrichment in NSCs

**Figure 2:**
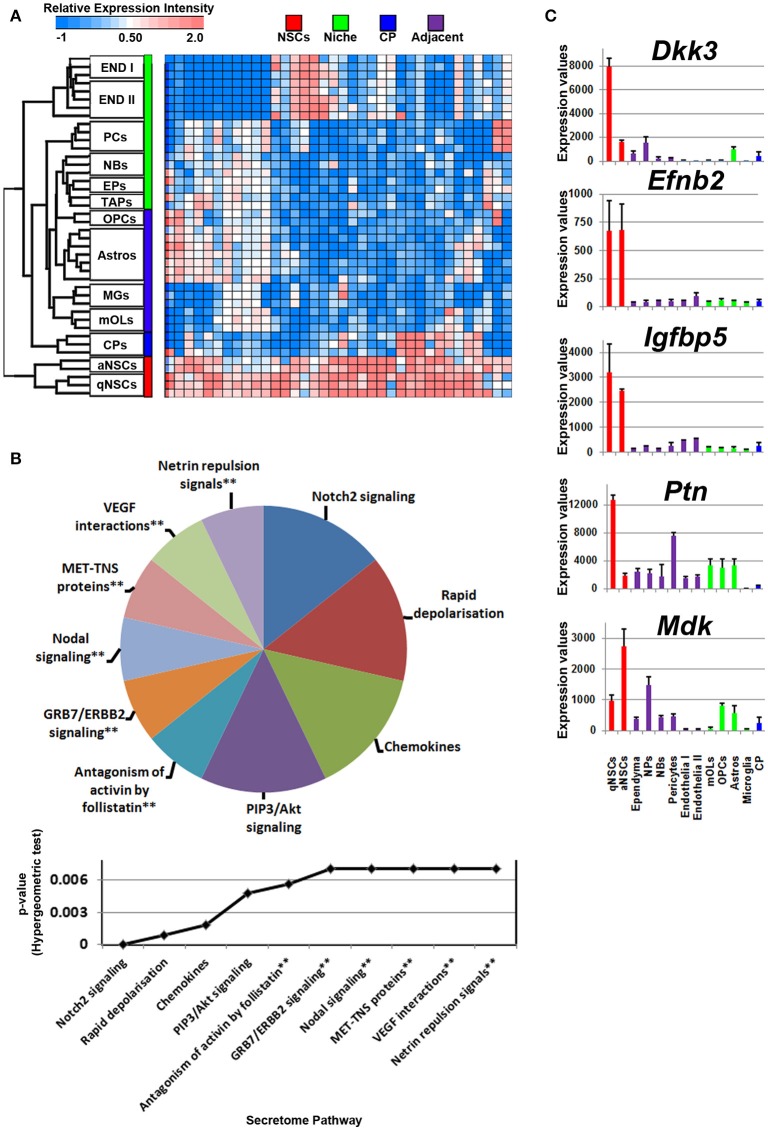
Autocrine/paracrine ligand mRNA enrichment in NSCs. **(A)** The transcriptome of significantly enriched (1.8 fold change; *p* < 5% FDR) ligands in both qNSCs and aNSCs are displayed as a heatmap and plotted with the remaining datasets. Some ligand genes show similar or lower mRNA enrichment with other cell types. **(B)** These were further characterized using Reactome Pathway for classifying broadly the functions and signaling networks of ligand mRNA detected in the analysis. The top 10 pathways were selected and ranked clockwise by their *p*-values and plotted below as a histogram. Note: double asterisks signify only 1 ligand mRNA associated within the pathway. **(C)** The top most enriched genes are shown in histograms with in their antilog expression values across multiple cell types showing overall elevated expression in NSCs. Red, green, blue and purple colors used are for groups containing NSCs, niche, choroid plexus and adjacent glial cells respectively. CP, choroid plexus; aNSCs, active neural stem cells; qNSCs, quiescent neural stem cells; MGs, microglia; OPCs, oligodendrocytes precursor cells; Astros, astrocytes; OLs, oligodendrocytes (mature); NBs, neuroblasts; TAPs, transiently amplifying progenitors; EPs, ependymal; PCs, pericytes; END I, Endothelia 1; END II, Endothelia 2.

Compared with all other groups, NSCs expressed relatively few ligands. Such ligands are shared in expression between other cell types analyzed including their downstream progenitors, TAPs and NBs, which is unlike the expression patterns of transcription factors that are shared between progenitors and NSCs (Azim et al., 2015). The small proportion of ligands enriched in NSCs implies that in the context of normal adult differentiation and maturation, NSCs appear to be mainly supported by external cues rather than providing direct stimuli to their environment in an auto- or paracrine manner. This is due to NSCs expressing relatively fewer transcripts compared to other sources or regions. Some transcripts were found to be highly abundant in NSCs compared to other cell types, with few transcripts shared between endothelia and astrocytes (Figure 2A). The secretome analysis was used to identify which signaling pathways are associated with the ligand transcripts detected in NSCs and arranged by their significance. This revealed pathways associated with Notch2 signaling, rapid depolarization (i.e., *Fgfs*), chemokines and ligands that stimulate Akt signaling via PIP3 (*Hgf, Nrg2*). The remaining pathways were narrowly significant due to the lower number of ligand genes expressed in NSCs (Figure 2B). The top five most highly enriched transcripts in NSCs in this analysis were *Dkk3, Efnb2, Igfbp5 Ptn*, and *Mdk* with most of them being uniquely expressed in NSCs compared to other cell types sampled (Figure 2C). *Dkk3* and *Efnb2* have been described previously as being enriched in the walls of the SVZ (Diep et al., 2004) as well as in a high-to-low expression gradient from the wall to the striatum (Ottone et al., 2014), respectively, thus confirming our findings. Most ligands were homogeneous to NSCs, whereas some were enriched in either qNSCs or aNSCs (highlighted in Supplementary Figure 1). Additional ligands which were not pursued further included *Hgf, Fgf13, Jag1, Fgf11, Vegfa, Bmp1, Inhbb*, that are likely to control neurogenesis/gliogenesis in an autocrine/paracrine manner (Supplementary Figure 1).

### Signaling ligand enrichment in glial cells of adjacent tissues

**Figure 3:**
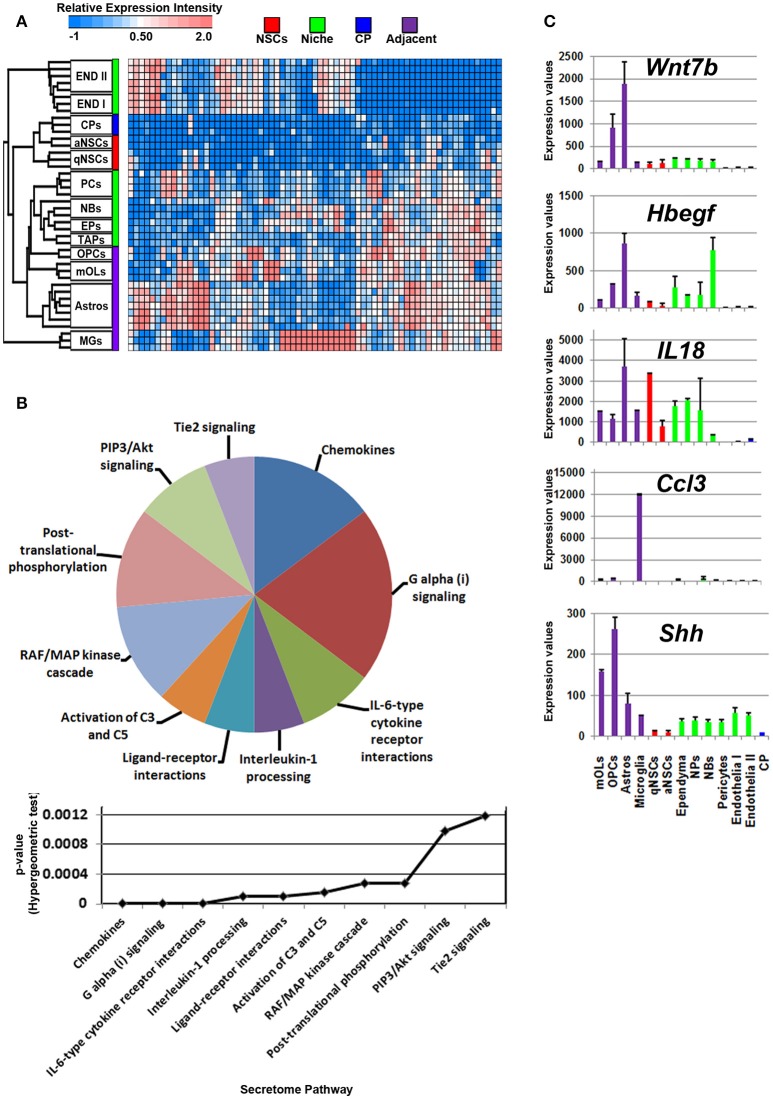
Enrichment of ligand mRNA derived from adjacent glial cells. **(A)** The transcriptome of ligands significantly (1.8 fold change; *p* < 5% FDR) enriched in subpopulations of glial cells are displayed as a heatmap and plotted with the remaining datasets. Ligands enriched in MGs are highly expressed in these cells compared to other cells types whereas those in astrocyte and oligodendroglia show some exclusiveness in their expression and some shared expression with other cells. **(B)** These were further characterized using Reactome Pathway for classifying broadly the functions and signaling networks of ligand mRNA detected in the analysis and their *p*-values plotted below as a histogram. **(C)** The top most enriched genes are shown in histograms with in their antilog expression values across multiple cell types showing overall elevated expression in different glial cells. Red, green, blue and purple colors used are for groups containing NSCs, niche, choroid plexus and adjacent glial cells, respectively. CP, choroid plexus; aNSCs, active neural stem cells; qNSCs, quiescent neural stem cells; MGs, microglia; OPCs, oligodendrocytes precursor cells; Astros, astrocytes; OLs, oligodendrocytes (mature); NBs, neuroblasts; TAPs, transiently amplifying progenitors; EPs, ependymal; PCs, pericytes; END I, Endothelia 1; END II, Endothelia 2.

Glial cells in the vicinity of the SVZ lateral and dorsal walls will contribute to neurogenesis/gliogenesis as previously demonstrated for astrocytes, oligodendrocytes and microglia (reviewed in Morrens et al., 2012; Su et al., 2014). Probes significantly enriched in glial cells were plotted on a heatmap revealing overlaps between OLs, OPCs, and astrocytes with cells in the niche. Of note, microglia expressed a number of unique ligands and clustered at a distance compared to other glial cells that were similar in their proximities (Figure 3A). According to the secretome analysis, at least half of the major pathways associated with glial ligands resembled immune-like signaling events via the detected chemokine and interleukin transcripts whereas some ligands point to common proliferative and survival functions via pathways involving Akt, RAF/MAP kinases. For example, *Angpt1, Fgf1, Hbegf*, and *Hgf* genes were upregulated in these cells, suggesting that some promote EGF-like functions (Figure 3B, Supplementary Figure 2). Within the pathway “ligand-receptor interactions,” *Hhip* and *Shh* that are enriched in oligodendroglia appeared highly significant. Ligands (*C4b, Chrdl1, Csf1, Il6)* which contribute to IGF regulation, transport, uptake by Igfbps, i.e., sequestering *Igfs* by *Igfbps*, imply some mode of trophic factor modulation. Overall, *Wnt7b, Hbegf, IL18, Ccl3*, and *Shh* are the top five major ligands enriched in adjacent glial cells (Figure 3C), deciphering novel highly enriched transcripts involved in glial to niche/NSC signaling.

### Signaling ligand enrichment in the choroid plexus

**Figure 4:**
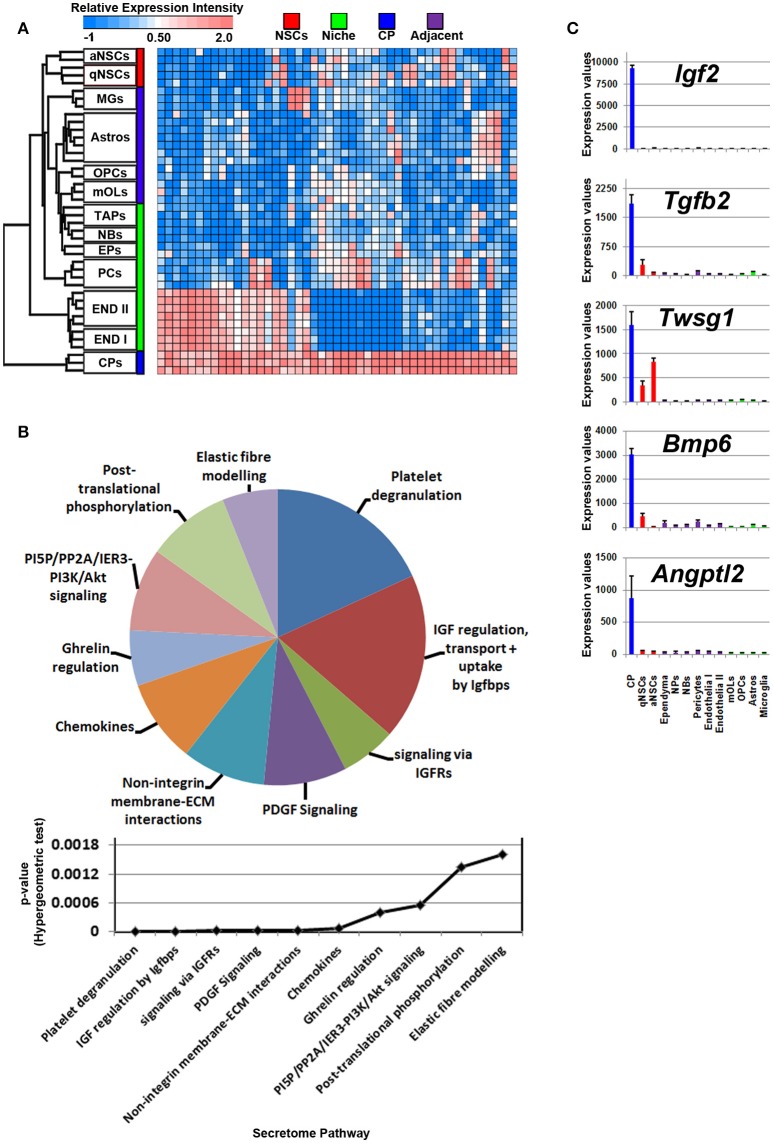
Ligand mRNA enrichment in the CP. **(A)** The transcriptome of ligand mRNA significantly enriched (1.8 fold change; *p* < 5% FDR) in the CP are displayed as a heatmap and plotted with the remaining datasets. Some ligand genes are abundant in the CP with some shared expression in vasculature. **(B)** These were further characterized using Reactome Pathway for classifying broadly the functions and signaling networks of ligand mRNA detected in the analysis and their *p*-values plotted below as a histogram. **(C)** The top most enriched genes are shown in histograms with in their antilog expression values across multiple cell types showing overall elevated expression in the CP. Red, green, blue and purple colors used are for groups containing NSCs, niche, choroid plexus and adjacent glial cells, respectively. CP, choroid plexus; aNSCs, active neural stem cells; qNSCs, quiescent neural stem cells; MGs, microglia; OPCs, oligodendrocytes precursor cells; Astros, astrocytes; OLs, oligodendrocytes (mature); NBs, neuroblasts; TAPs, transiently amplifying progenitors; EPs, ependymal; PCs, pericytes; END I, Endothelia 1; END II, Endothelia 2.

The cerebral spinal fluid (CSF) is formed mainly by the choroid plexus (CP), an epithelial-like structure present internally around the brain ventricles that expresses and secretes a number of signaling ligands throughout life which is why this structure is considered an important regulator of development, differentiation and maturation (Silva-Vargas et al., 2016). Ligands enriched in the CP were plotted on a heatmap (Figure 4A). Consistent with their morphology and classification as mural cells, a subset of signaling ligands were found to overlap in expression with those deriving from the vasculature (Figure 4A), therefore suggesting that many ligands enriched in expression in the CP are also synthesized by endothelia and pericytes. The secretome pathway analysis revealed some “pro-neurogenic” terms as being upregulated and specific to CP such as signaling via IGFRs, non-integrin membrane-ECM interactions and PI5P/PP2A/IER3-PI3K/Akt. A number of terms which emerged as being significant in the analysis were due to the expression of *Igfs* and *Igfbps* that were vastly abundant in the CP (Figure 4B). Of note, the top five CP enriched ligands are *Igf2, Tgfb2, Twsg1, Bmp6*, and *Angptl2* (Figure 4C) and were almost exclusively expressed in the CP. Other ligands with relative abundant expression in the CP include *Fgf2, Gas6, Pdgfa, Pdgfd, Tgfb2, Bmp7*, all of which have previously been confirmed elsewhere as major neurogenic determinants (Marques et al., 2011; Supplementary Figure 3).

### Signaling ligand enrichment in the niche

The PCA described earlier showed that endothelia are distinct from any other cell type included in the analysis whereas the others showed some degree of relatedness (Figure 1A). Accordingly, the niche that consists of progenitors, neuroblasts, ependymal- and cells of the vasculature should contain the largest cohort of signaling ligand transcripts. The analysis was done by incorporating datasets of endothelial cells derived from two different studies as a control and as a consequence essentially the same expression patterns were observed (see Materials and Methods). Of note, datasets of niche-specific astrocytes (also termed as B2 astrocytes), a smaller proportion of cells in the niche that may also contribute to neurogenesis/gliogenesis (Platel and Bordey, 2016), are not yet available in any platform.

**Figure 5:**
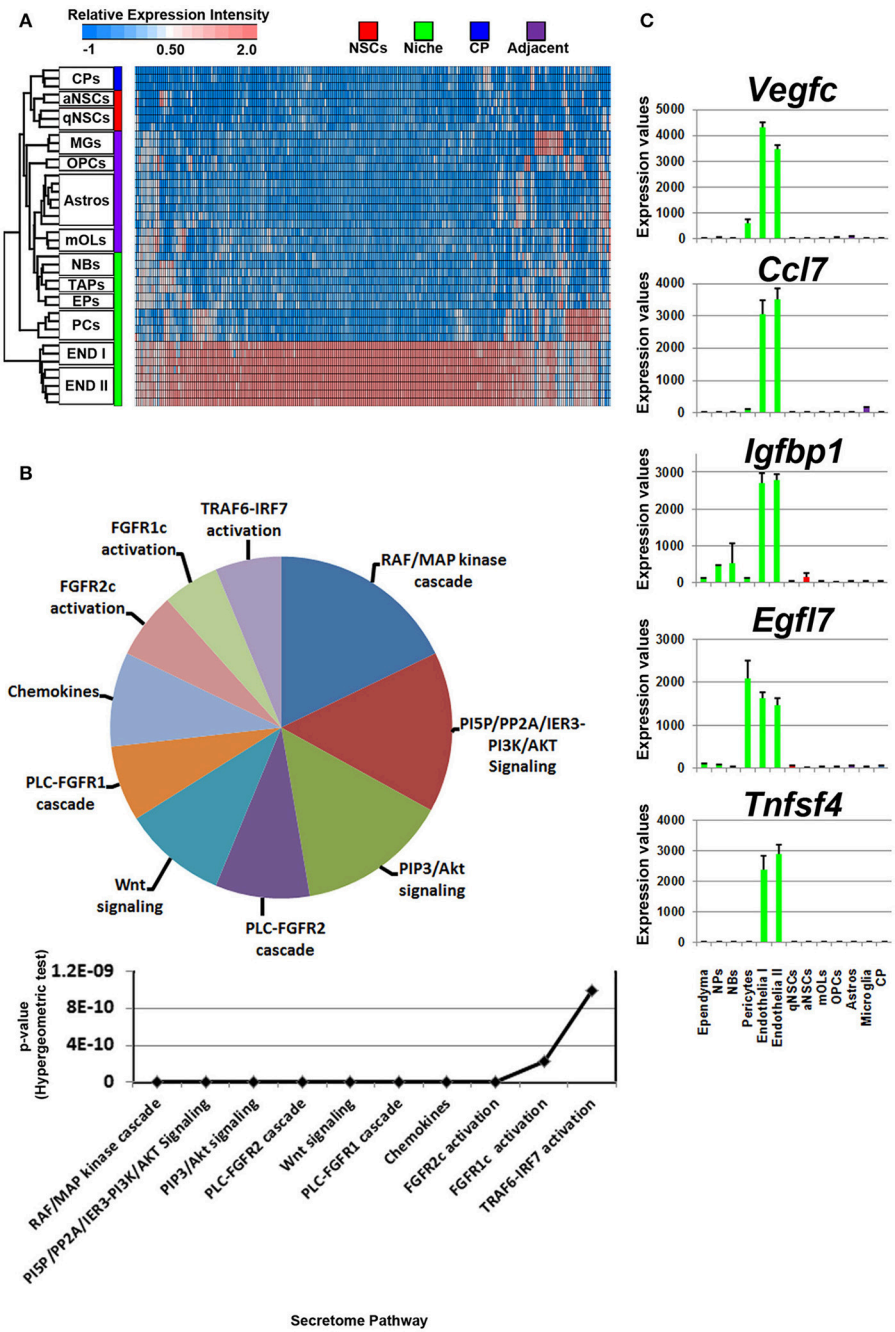
Ligand mRNA enrichment in cells that constitute the niche**. (A)** The transcriptome of ligand mRNA significantly enriched (1.8 fold change; *p* < 5% FDR) in the cells derived from the niche are displayed as a heatmap and plotted with the remaining datasets. The majority of ligand mRNA detected in this analysis is derived from endothelia. **(B)** These were further characterized using Reactome Pathway for classifying broadly the functions and signaling networks of ligand mRNA detected in the analysis and their *p*-values plotted below as a histogram. **(C)** The top most enriched genes are shown in histograms with in their antilog expression values across multiple cell types showing overall elevated expression in different glial cells. Red, green, blue and purple colors used are for groups containing NSCs, niche, choroid plexus and adjacent glial cells, respectively. CP, choroid plexus; aNSCs, active neural stem cells; qNSCs, quiescent neural stem cells; MGs, microglia; OPCs, oligodendrocytes precursor cells; Astros, astrocytes; OLs, oligodendrocytes (mature); NBs, neuroblasts; TAPs, transiently amplifying progenitors; EPs, ependymal; PCs, pericytes; END I, Endothelia 1; END II, Endothelia 2.

Indeed, most of the ligands detected were expressed in the niche (Figure 1B) and moreover, as anticipated, the vast majority of ligands were abundantly expressed in endothelia (Figure 5A). Furthermore, some ligands were partly shared with other cell types (white or red hot spots of Figure 5A) but the overall majority of ligands were unique in expression to endothelia, slightly overlapping with pericytes. Altogether, the niche itself and particularly endothelia display the most abundant expression of signaling ligands, although the extent of their functionality in neurogenesis is not understood.

The secretome pathway analysis revealed only a few overlapping pathways as seen for NSCs, glial cells and the CP. Aside from *Fgf2* which was highly abundant in the CP, the 10 other members of the FGF-family of proteins were enriched in the niche, which resulted in several different pathways associated with Fgf-signaling (Figure 5B). For the first time, a pathway enrichment for “Wnt signaling” was also evident since several Wnt mRNAs are expressed in endothelia. This enrichment analysis also revealed a variety of other pro-neurogenic pathways that were lowly ranked but still emerged as significant such as signaling by BMP, ERBB2, glycoprotein hormones, or via Eph-Ephrin interactions. Very few inhibitory pathways were observed from endothelia, with the notable exception of “negative regulation of TCF-dependent signaling by WNT ligand antagonists,” suggesting an overall trophic nature of niche derived signals (Figure 5B). The top five ligands detected in the niche were almost exclusively derived from the vasculature (Figure 5C) and corresponded to *Vegfc, Ccl7, Igfbp1, Egfl7*, and *Tnfsf4*. The top 5 most enriched ligands were ranked into their respective known ligand families and the top 10 most abundant family members are presented in Table 1 for their first glance preview. Here, a number of ligands from the same families have overlapping functions for example, *Wnt7a* and *Wnt8b* (Choe et al., 2015), and multiple members of the Fgf-family of ligands (Ornitz and Itoh, 2015).

**Table 1:** Ligands enriched in the niche were classified by the signaling families they are grouped in.

**Immune Related**			**Wnts**		
**Gene symbol**	**Fold change**	***p-*value**	**Gene symbol**	**Fold change**	***p-*value**
*Tnfsf4*	10.8	3.1E−08	*Wnt5a*	8.0	8.2E−13
*C1qtnf4*	7.7	2.6E−06	*Wnt7a*	5.8	1.3E−18
*Ifna11*	4.9	2.2E−07	*Frzb*	4.8	7.9E−07
*C1ra*	4.7	3.4E−05	*Wnt8b*	4.7	2.4E−06
*Ifng*	4.5	3.5E−07	*Rspo2*	2.9	4.0E−07
**BMP/TGFβ**			**Interleukins**		
**Gene symbol**	**Fold change**	***p-*****value**	**Gene symbol**	**Fold change**	***p-*****value**
*Grem2*	8.6	2.7E−07	*Il7*	5.4	3.7E−06
*Tgfb3*	6.3	1.4E−09	*Il34*	4.2	3.9E−13
*Gdf11*	4.8	4.1E−08	*Il1a*	3.7	5.2E−04
*Nog*	4.3	2.1E−05	*Il23a*	3.4	3.7E−03
*Bmp5*	4.2	4.3E−07	*Il1f5*	3.3	9.4E−09
**Chemokines**			**FGFs**		
**Gene symbol**	**Fold change**	***p-*****value**	**Gene symbol**	**Fold change**	***p-*****value**
*Ccl7*	17.1	4.1E−10	*Fgf6*	5.7	1.4E−07
*Cxcl5*	3.8	1.4E−06	*Fgf3*	5.6	8.4E−07
*Ccl11*	3.6	4.5E−07	*Fgf18*	5.3	6.4E−05
*Cxcl3*	3.5	2.7E−06	*Fgfl*	4.6	3.4E−03
*Ccl5*	3.1	2.3E−06	*Fgf4*	4.4	9.2E−09
**Prolactins**			**EGF-Iike**		
**Gene symbol**	**Fold change**	***p-*****value**	**Gene symbol**	**Fold change**	***p-*****value**
*Prl2c2*	8.5	1.4E−07	*Edil3*	5.1	8.7E−08
*Pr l2a1*	5.5	3.1E−08	*Epgn*	4.4	3.3E−08
*Pr l2b1*	5.2	4.3E−08	*Vwce*	3.3	2.4E−07
*Pr l3d1*	4.8	1.8E−09	*Btc*	3.0	1.3E−04
*Prl7a2*	4.6	5.1E−08	*Nrg2*	2.3	2.2E−12
**Hormone-like**			**IGF family and regulation**		
**Gene symbol**	**Fold change**	***p-*****value**	**Gene symbol**	**Fold change**	***p-*****value**
*Grp*	5.4	4.5E−06	*lgfbpl1*	15.3	5.0E−08
*Adm*	4.4	2.2E−06	*lnsl*	9.8	8.8E−16
*Gcg*	4.2	9.1E−07	*lgfbp4*	6.1	1.2E−04
*Gast*	3.9	7.8E−06	*lgfl*	5.4	1.0E−05
*Tshb*	3.8	7.4E−10	*lgfbpl*	4.1	1.1E−07

*The top 10 most abundant ligand families are summarized and each ligand per family is ranked in order from high-to-low. The top 5 ligands per group are presented*.

Ligands enriched in ependymal or NPs were overall very few, implying that these cells expressed fewer ligands. Another important neurogenic ligand, *Egf*, of which the expression was higher in TAPs and neuroblasts, although likely resulting from the large variability within these datasets, was not significantly changed. Similarly, the expression of *Cntf* was high in TAPs, neuroblasts and astrocytes, but only in astrocytes the expression was stable among the replicates. Generally, with the inclusion of endothelia and pericytes in the analysis, very few ligands show any enrichment in neuroblasts or TAPs or the entire niche vs. the other region/cell types demonstrating that the vasculature contains abundant sources of ligand mRNA (see also Supplementary Figure 4).

## Discussion

Much of the information regarding ligand expression in adult neurogenic niches has been yielded from classical *in situ* experiments which provided a gross overview on their expression at the anatomical level. The ever increasing availability of cell purified WGT datasets in GEO enables thorough meta-analyses to be performed for identifying gene expression patterns of interest. Such an approach is relevant for understanding the basic biology behind upstream developmental/neural turnover purposes and could therefore facilitate target identification for therapeutic intervention.

Here, we present a thorough signaling ligand expression analysis across a variety of cell types present in neurogenic niches, glial cells and choroid plexus. Identified ligand patterns were then correlated with information on matching receptor expression taking into account that multiple receptors for each family of ligands can exist (for example the >25 known receptors for Wnt ligands that are spatiotemporally expressed in the SVZ; Harrison-Uy and Pleasure, 2012). Nevertheless, exposed ligand/receptor couples may reveal important, currently unrecognized signaling events contributing to adult neuro- and gliogenic activities. The nature of the analysis performed, i.e., examining region-specific hallmarks within multiple datasets, allowed the detection of transcripts with a degree of specificity rather than transcripts that are shared in expression broadly across a variety of cell types.

**Figure 6:**
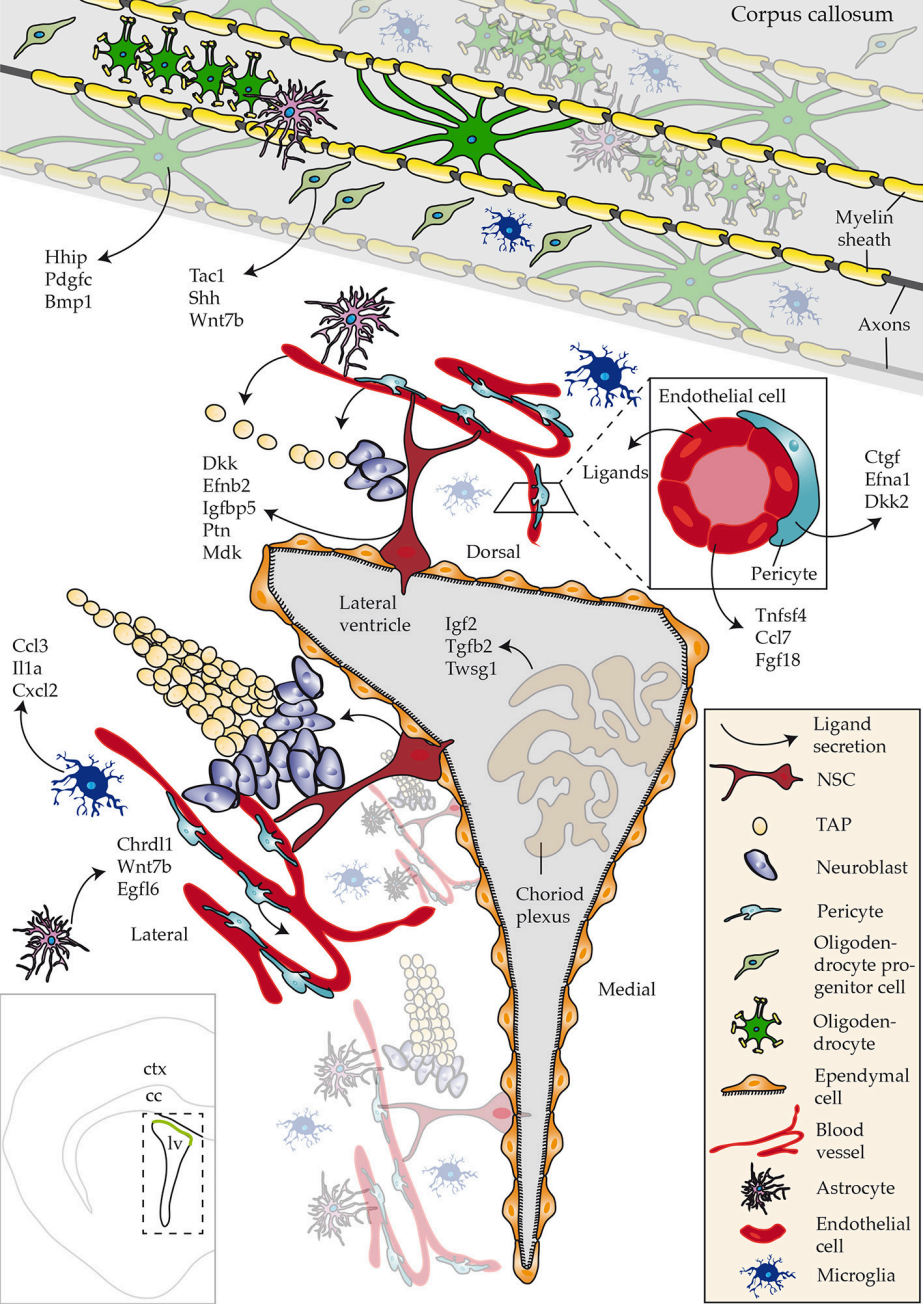
Schematic overview of ligands identified that regulate adult neurogenesis. An overview of ligands detected with a focus on novel ligand expression in studied regions and cell types. Bottom left hand corner inset shows an overview of a coronal section of the brain highlighting the lateral ventricle (lv), corpus callosum (cc) and cortex (ctx).

The goal of this study was to describe enrichment of ligands in defined regions or subpopulations of cells in close proximity to the walls of the SVZ that regulate aspects of neurogenesis/gliogenesis. Datasets used in the study were derived from acutely isolated cells, using as similar procedures as possible, thereby eliminating any potential false-positives. Future studies involving single cell sequencing of all known cell types that are present close to neurogenic regions will address any potential caveats. To date, such a resource is available but lacks datasets of cells constituting the niche, i.e., NSCs, progenitors (Zeisel et al., 2015). However, the results also indicate that cocktails of ligands triggering multiple intracellular signaling networks might be operating during the distinctive steps of NSC differentiation–a notion that should be considered for future functional studies. Our key findings of the most abundant ligands identified in CNS regions or cell types are summarized in Figure 6.

### The vasculature constitutes an important determinant of neurogenic processes

A novel finding in this study was the identification of over 200 ligand mRNAs enriched in cells that form the niche, whilst the bulk of these transcripts are attributed to endothelia in contrast to ependymal cells, TAPs and neuroblasts. This might partially result from the little information available regarding ligand expression in ependymal cells, TAPs or neuroblasts in the adult SVZ which is currently limited to a combined number of 10 transcripts. In this regard it is of interest to note that for example *Nog*, a Bmp inhibitor previously reported to be expressed in mainly ependymal cells (Lim et al., 2000), has in the present study been found to be abundant in endothelia with low expression in any other cell type. *Efnb3* encoding the Ephrin-B3 ligand binding to Eph tyrosine kinase receptors revealed to be expressed in both neuroblasts and ependymal cells, a finding confirming the patterns of expression at the protein level (Theus et al., 2010). In another study sampling postnatal neuroblasts close to the walls of the SVZ and those migrating to the olfactory bulb, also only few ligands have been identified (Khodosevich et al., 2009), similar to the adult SVZ-neuroblasts examined here.

Endothelia expressed virtually all known major pathway ligands and several members of any given family. This included at least 18 members of prolactins, 18 chemokines, 12 Fgfs, at least 11 Wnt ligands (canonical and non-canonical), and 24 Bmp/TGFβ-related transcripts. On the other hand, many of the ligands enriched in pericytes overlap with those of endothelia or correspond to related family members, suggesting a certain degree of redundancy among endothelia and pericytes. Inhibitory-like ligands were broadly classified as “ligands” in both endothelia and pericytes, and only appeared at relatively low percentages, thus, demonstrating the large extent of trophic support from the vasculature.

Of note, the used WGT datasets of endothelia or pericytes were suboptimal as SVZ specific expression profiles are currently not available, so that the datasets analyzed in this study were derived from the adult cortex (Nolan et al., 2013; Coppiello et al., 2015). It is anticipated that some degree of heterogeneity of the vasculature in different CNS compartments exists. However, analysis of endothelia purified from the cortex, heart and liver have not reported any marked differences in ligand expression, in contrast to transcriptional regulators that are differentially expressed in endothelia of different origins (Coppiello et al., 2015). Information regarding endothelial heterogeneity in the CNS is restricted to a few studies that show *Vegfa, Vegfb*, and *App* appearing to be expressed at similar levels among endothelia from SVZ and cortex (Crouch et al., 2015; Sato et al., 2017). The absence of SVZ specific datasets is even further deplorable, as it has been suggested that the cortical vasculature is non-neurogenic, assumingly due to astrocytic end-feet interactions with blood vessels (Ninkovic and Götz, 2013).

Still, the finding that over 200 ligands enriched exclusively in endothelia and pericytes, is intriguing and supports earlier observations on endothelia of non-neurogenic as well as neurogenic origins shown to enhance aNSC proliferation compared to qNSCs and TAPs *in vitro* (Crouch et al., 2015). At the same time, specific ligands such as Jagged1- and EphrinB2-signaling derived from the vasculature were shown to maintain NSC quiescence (Ottone et al., 2014). Multiple regulatory modes on neurogenesis/gliogenesis are likely to occur and await further functional description. In this regard spatio-temporal combinations and synergistic effects will have to be taken into account.

### Cell intrinsic environmental cues that maintain NSCs

Neural stem cells (NSCs) during postnatal or embryonic development express fewer transcripts for ligands compared to adult NSCs, (Azim et al., 2015). It has to be mentioned that the NSC datasets analyzed here are mainly derived from the lateral wall whereas those originating from the dorsal wall are likely to show additional heterogeneity in ligand expression as described for region specific postnatal NSCs (Azim et al., 2015). Despite these limitations, the profiles of a few key candidates were obtained. For example, the abundant expression of the Wnt pathway antagonist, *Dkk3* in qNSCs implies a broad autocrine or paracrine mode of negative regulation in neurogenesis/gliogenesis (Zhu et al., 2014), and, presumably with other Wnt antagonists such as *Srfp1* that is also highly expressed in qNSCs, represses dorsal SVZ identities. Similarly, the chemoattractant *Ptn* has recently been described in drawing glioma cells toward the SVZ prior to further invasion (Qin et al., 2017). Although poorly studied in terms of neurogenic/gliogenic functionality, its elevated expression in qNSCs signifies that it may guide other cells in the vicinity of qNSCs for further trophic support.

### Cues deriving from adjacent glial cells that may regulate NSC behavior

Subpopulations of glial cells close to SVZ walls contribute to NSC behavior (reviewed in Morrens et al., 2012; Su et al., 2014). The secretome pathway analysis showed that many of the glial-derived ligands resemble “inflammatory,” “chemokine,” or “immune-like” pathways consistent with known micro- and astroglial expression signatures (Ribeiro Xavier et al., 2015). It is anticipated that some of these ligands will have pro-neurogenic functions, whereas others such as for example *IL18* may impair neurogenesis/gliogenesis (Crampton et al., 2012). Overall, the immune-related nature of the bulk of these ligands as we are used to know them may have to be reconsidered as they appear to exert non-immune related functions in the physiologically normal brain. Other detected ligands such as TNFα and IFNβ have been shown to promote oligodendrogenesis at the expense of neurogenesis (Tepavcevic et al., 2011; reviewed in Covacu and Brundin, 2015).

### Long distance-regulation of neurogenic processes by the CP

The CP has long been regarded as an important source of ligand secretion into the CSF amongst its well-known functions in maintaining CSF constituents. It is noteworthy that the protruding cilia of NSCs directly contact the CSF in regulating NSC behavior (reviewed in Lun et al., 2015) and that ligands derived from the CP guide neuroblast migration by widespread dispersion into the SVZ tissue via the CSF flow (Sawamoto et al., 2006). Therefore, ligands secreted from the CP will not only affect NSCs, but also cells close to the lateral ventricle walls. One aim of the present study was to determine the expression profiles of ligand mRNA in the CP compared to other cell types sampled. This would define which expressed ligands are relatively unique to the CP and which are only additionally expressed by the CP as a supplementary source confirming their known expression patterns elsewhere (Marques et al., 2011). Furthermore, by sampling a number of other WGT datasets, at least these analyzed ligand mRNA expression profiles are uniquely confined to the CP. A large number of CP ligands are also shared in expression with their mural counterparts, endothelia and pericytes such as for example *Bmp5, Bmp7* and *Fgf*, implying a “surplus” mode of ligand generation.

## Conclusion

In the present study, we could address the unsolved issue of ligand expression in the major neurogenic niche of the forebrain, the SVZ. Our results have unraveled an unexpected level of diversity and complexity in ligand expression that is likely to orchestrate the distinct neurogenic processes such as survival, proliferation, maintenance, and fate acquisition. The key findings based on the datasets used in this study imply that the niche is an important source of signaling regulation. Some other ligands could additionally be engaged in long-range communication, particularly those emanating from the CP to cells that reside in the niche acting as a means for further regulating aspects of NSCs. As many of the here described novel ligands have not yet been tested for functionality on NSCs, future studies are warranted not only in regard of matching receptor profiles but also in terms of neurogenic and/or gliogenic functions. In the case of neurogenesis in the human SVZ, it will be interesting to compare human-derived datasets of NSCs and niche components. This would enable focusing on novel ligands common to both species for functional studies in mouse in order to eventually be used for therapeutical interventions in humans. Moreover, the data presented here will serve as a guidepost for future studies on related cell populations following disease or in ageing.

## Author contributions

KA: conceptualization, data curation, formal analysis, funding acquisition, investigation, methodology, project administration, supervision; RA: writing, data curation, formal analysis, investigation, methodology; MC: data curation, formal analysis, funding acquisition, investigation, methodology; JJ: writing, visualization, investigation, methodology; JV: funding acquisition, project administration, supervision; PK: funding acquisition, investigation, methodology, project administration, supervision, validation, writing.

### Conflict of interest statement

The authors declare that the analysis performed in the absence of any commercial or financial relationships that could be construed as a potential conflict of interest. The handling Editor and reviewer AB declared their involvement as co-editors in the Research Topic, and confirm the absence of any other collaboration

## Supplementary material

Supplementary Figure 1 A transcriptomic map of ligand expression in NSCs. All transcripts enriched in NSCs across multiple cell types presented as a heatmap. Red, white and blue are relative intensity expression of 2, 0.5, and −1 respectively. Gene symbols in red, light red and black show enrichment (≥1.8 fold change; ≤ 5% FDR) in qNSCs, aNSCs or homogenous expression to both subpopulations. CP, choroid plexus; aNSCs, active neural stem cells; qNSCs, quiescent neural stem cells; MGs, microglia; OPCs, oligodendrocytes precursor cells; Astros, astrocytes; OLs, oligodendrocytes (mature); NBs, neuroblasts; TAPs, transiently amplifying progenitors; EPs, ependymal; PCs, pericytes; END I, Endothelia 1; END II, Endothelia 2.

Supplementary Figure 2 A transcriptomic map of ligand expression in adjacent glial cell**s**. All transcripts enriched in glial cells across multiple cell types presented as a heatmap. Red, white and blue are relative intensity expression of 2, 0.5, and −1 respectively. CP, choroid plexus; aNSCs, active neural stem cells; qNSCs, quiescent neural stem cells; MGs, microglia; OPCs, oligodendrocytes precursor cells; Astros, astrocytes; OLs, oligodendrocytes (mature); NBs, neuroblasts; TAPs, transiently amplifying progenitors; EPs, ependymal; PCs, pericytes; END I, Endothelia 1; END II, Endothelia 2.

Supplementary Figure 3 A transcriptomic map of ligand expression in the choroid plexus. All transcripts enriched in the CP across multiple cell types presented as a heatmap. Red white and blue are relative intensity expression of 2, 0.5 and −1 respectively. CP, choroid plexus; aNSCs, active neural stem cells; qNSCs, quiescent neural stem cells; MGs, microglia; OPCs, oligodendrocytes precursor cells; Astros, astrocytes; OLs, oligodendrocytes (mature); NBs, neuroblasts; TAPs, transiently amplifying progenitors; EPs, ependymal; PCs, pericytes; END I, Endothelia 1; END II, Endothelia 2.

Supplementary Figure 4 A transcriptomic map of ligand expression in the niche. The top 100 transcripts enriched in cells that constitute the niche across multiple cell types presented as a heatmap. Red, white and blue are relative intensity expression of 2, 0.5, and −1 respectively. CP, choroid plexus; aNSCs, active neural stem cells; qNSCs, quiescent neural stem cells; MGs, microglia; OPCs, oligodendrocytes precursor cells; Astros, astrocytes; OLs, oligodendrocytes (mature); NBs, neuroblasts; TAPs, transiently amplifying progenitors; EPs, ependymal; PCs, pericytes; END I, Endothelia 1; END II, Endothelia 2.

## Abbreviations

SVZ: subventricular zone
NSCs: neural stem cells
TAPs: transiently amplifying progenitors
TFs: transcription factors
WGT: whole genome transcriptome
NPs: neural precursors
FDR: false discovery rate
PCA: principle components plots
qNSCs: quiescent NSCs
aNSCs: activated NSCs
CSF: cerebral spinal fluid
CP: choroid plexus
MGs: microglia
aNSCs: active neural stem cells
qNSCs: quiescent neural stem cells
Astros: astrocytes
OLs: oligodendrocytes (mature)
PCs: pericytes
END: endothelia.
