# Supplementary figures and images for: Transcriptional Profiling of Ligand Expression in Cell Specific Populations of the Adult Mouse Forebrain That Regulates Neurogenesis
Source: Front Neurosci. 2018 Apr 20;12:220. doi: 10.3389/fnins.2018.00220 (PMC5925963; doi:10.3389/fnins.2018.00220)

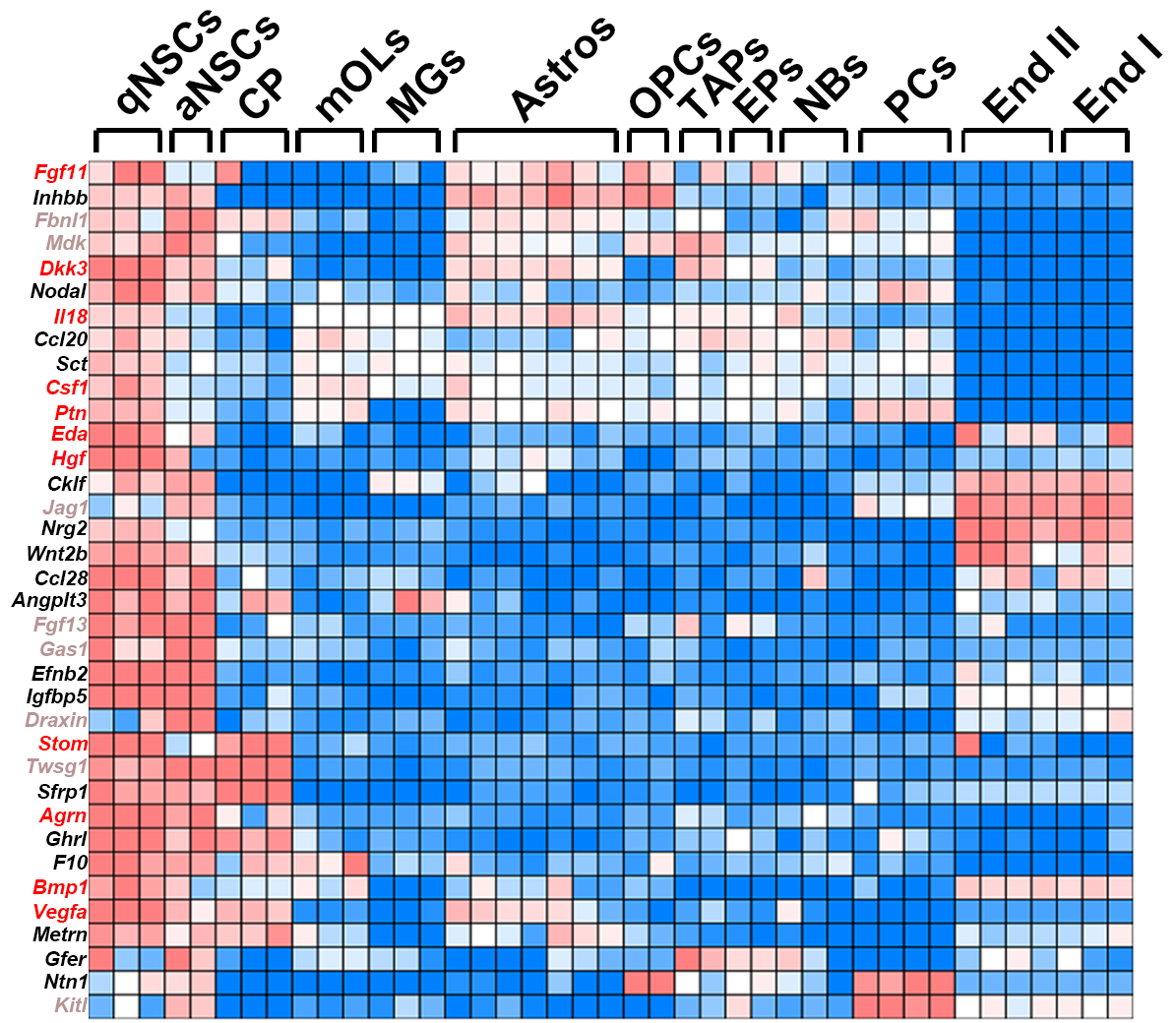

Supplement: Supplementary Figure 1 — A transcriptomic map of ligand expression in NSCs. All transcripts enriched in NSCs across multiple cell types presented as a heatmap. Red, white and blue are relative intensity expression of 2, 0.5, and −1 respectively. Gene symbols in red, light red and black show enrichment (≥1.8 fold change; ≤ 5% FDR) in qNSCs, aNSCs or homogenous expression to both subpopulations. CP, choroid plexus; aNSCs, active neural stem cells; qNSCs, quiescent neural stem cells; MGs, microglia; OPCs, oligodendrocytes precursor cells; Astros, astrocytes; OLs, oligodendrocytes (mature); NBs, neuroblasts; TAPs, transiently amplifying progenitors; EPs, ependymal; PCs, pericytes; END I, Endothelia 1; END II, Endothelia 2. [file Image1.TIF]

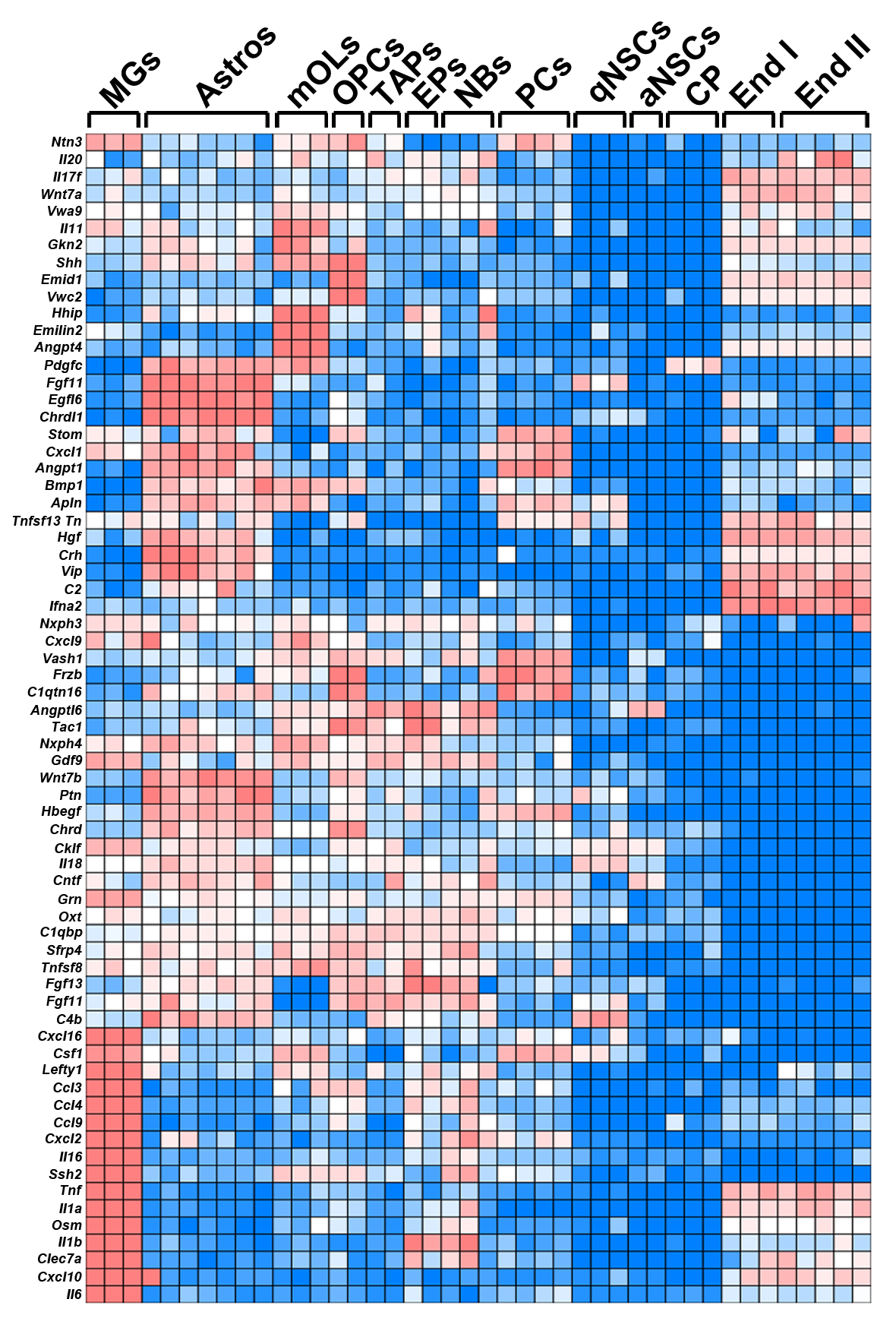

Supplement: Supplementary Figure 2 — A transcriptomic map of ligand expression in adjacent glial cells. All transcripts enriched in glial cells across multiple cell types presented as a heatmap. Red, white and blue are relative intensity expression of 2, 0.5, and −1 respectively. CP, choroid plexus; aNSCs, active neural stem cells; qNSCs, quiescent neural stem cells; MGs, microglia; OPCs, oligodendrocytes precursor cells; Astros, astrocytes; OLs, oligodendrocytes (mature); NBs, neuroblasts; TAPs, transiently amplifying progenitors; EPs, ependymal; PCs, pericytes; END I, Endothelia 1; END II, Endothelia 2. [file Image2.TIF]

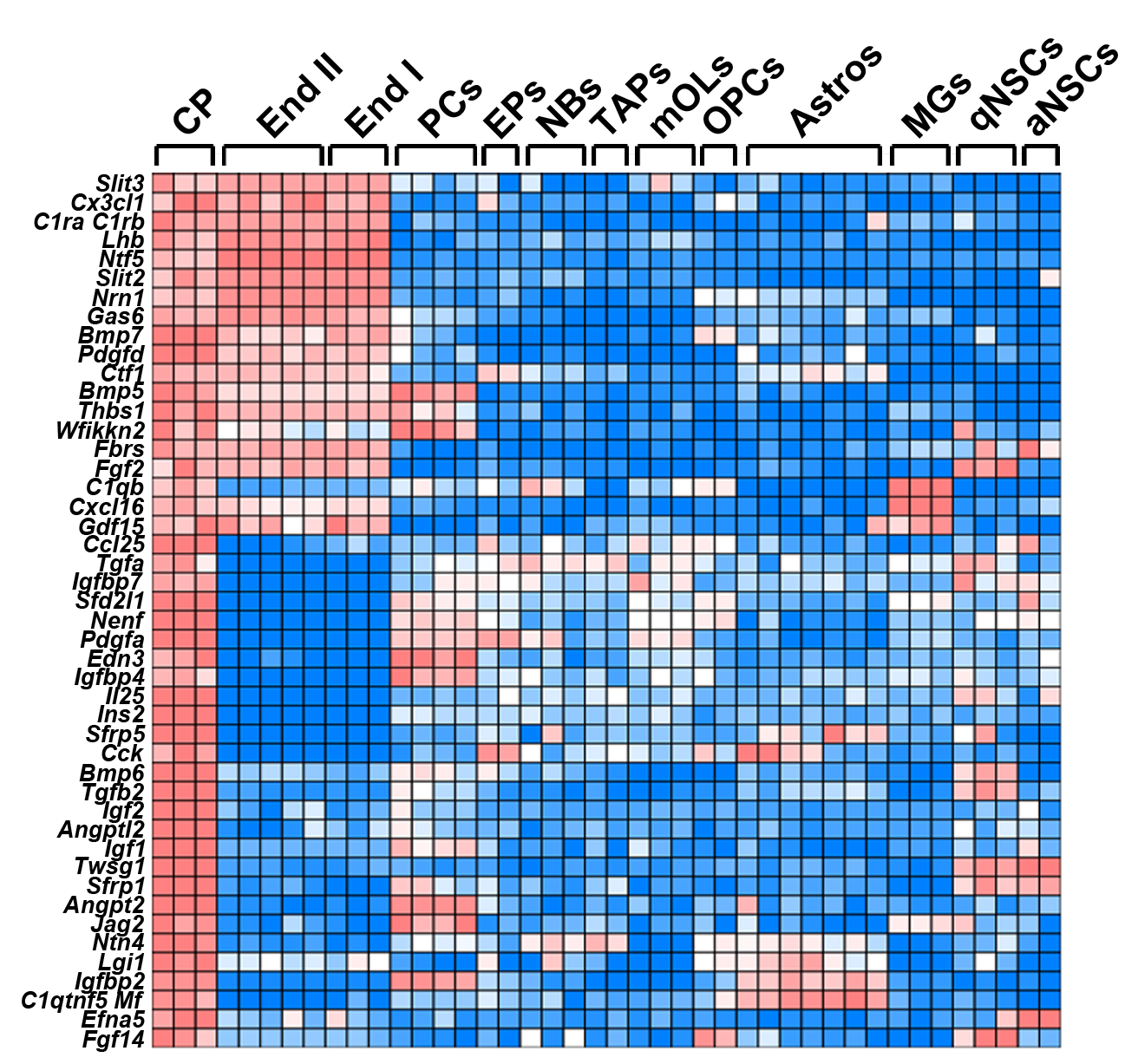

Supplement: Supplementary Figure 3 — A transcriptomic map of ligand expression in the choroid plexus. All transcripts enriched in the CP across multiple cell types presented as a heatmap. Red white and blue are relative intensity expression of 2, 0.5 and −1 respectively. CP, choroid plexus; aNSCs, active neural stem cells; qNSCs, quiescent neural stem cells; MGs, microglia; OPCs, oligodendrocytes precursor cells; Astros, astrocytes; OLs, oligodendrocytes (mature); NBs, neuroblasts; TAPs, transiently amplifying progenitors; EPs, ependymal; PCs, pericytes; END I, Endothelia 1; END II, Endothelia 2. [file Image3.TIF]

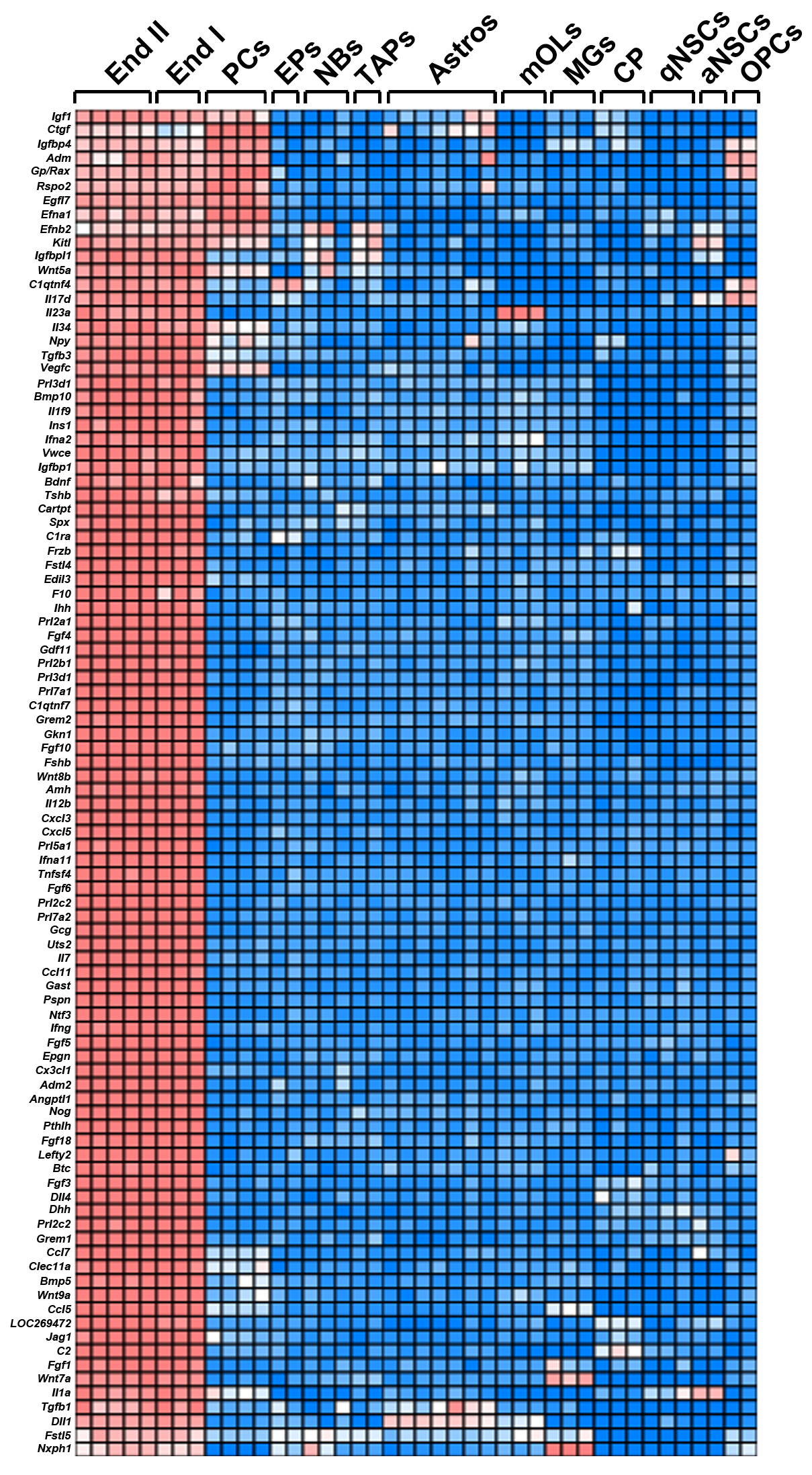

Supplement: Supplementary Figure 4 — A transcriptomic map of ligand expression in the niche. The top 100 transcripts enriched in cells that constitute the niche across multiple cell types presented as a heatmap. Red, white and blue are relative intensity expression of 2, 0.5, and −1 respectively. CP, choroid plexus; aNSCs, active neural stem cells; qNSCs, quiescent neural stem cells; MGs, microglia; OPCs, oligodendrocytes precursor cells; Astros, astrocytes; OLs, oligodendrocytes (mature); NBs, neuroblasts; TAPs, transiently amplifying progenitors; EPs, ependymal; PCs, pericytes; END I, Endothelia 1; END II, Endothelia 2. [file Image4.TIF]
